# Case report: subacute thyroiditis after receiving SARS-CoV-2 vaccine, maybe not only adjuvants

**DOI:** 10.3389/fmed.2022.856572

**Published:** 2022-08-05

**Authors:** Yizhou Huang, Xingyu Chen, Qing Wang, Xiaowei Lei, Lili Zhang

**Affiliations:** Department of Endocrinology, The Second Affiliated Hospital, Chongqing Medical University, Chongqing, China

**Keywords:** adverse vaccine reactions, subacute thyroiditis, SARS-CoV-2 vaccines, thyroid dysfunction, case report

## Abstract

Severe acute respiratory syndrome coronavirus 2 (SARS-CoV-2) induced the new coronavirus disease 2019 (COVID-19) pandemic worldwide. SARS-CoV-2 vaccines are designed to control the transmission of the disease. However, post-vaccination subacute thyroiditis (SAT) also appears with increase vaccination rate. Three cases of SAT after SARS-CoV-2 vaccines are described in this study. We have reported the patients’ clinical symptoms, laboratory tests, and thyroid imaging. Tests for COVID-19 were all negative, and the patients did not report thyroid-related diseases, autoimmune diseases, or preceding upper respiratory system infections in their medical history. Three female patients showed neck pain on physical examination. The laboratory test results and imaging findings were consistent with the diagnostic criteria of SAT. The patients were carried out a standardized treatment according to their symptoms, and we closely followed up their response to the treatment. Clinicians must be aware of the possibility of SAT after receiving the vaccines and make timely therapy.

## Introduction

The new coronavirus disease 2019 (COVID-19) pandemic, a devastating public health event, is caused by severe acute respiratory syndrome coronavirus 2 (SARS-CoV-2), with over 195 million reported cases and greater than 4.1 million deaths worldwide (1–3). The disease could cause pulmonary and systemic inflammations and has a broad spectrum of clinical manifestations, from asymptomatic to fatal respiratory damage (4). Meanwhile, data derived from several patients also demonstrated that it could lead to multiple organ dysfunction (5). However, it is noteworthy that the infection also does harm to the thyroid gland due to the highly expressed angiotensin-converting enzyme 2 (ACE2) protein, which was considered the initial entry for SARS-CoV-2, in the thyroid cells (6, 7). Specifically, SARS-CoV-2-related thyroid disorders include Graves’ disease, hypothyroidism, Hashimoto’s thyroiditis, thyrotoxicosis, and subacute thyroiditis (SAT) (6). SAT is a self-limiting thyroid inflammatory disease characterized by prominent clinical features, such as neck pain, fever, and thyroid dysfunction, and is accompanied by elevated erythrocyte sedimentation rate (ESR) and C-reactive protein (CRP) concentration (8, 9). Research has shown that SAT is usually followed by an upper respiratory tract infection and is strongly associated with many viruses (10). So far, emerging studies have illustrated that the coronavirus disease has close ties to SAT, and it has been reported that patients had symptoms of SAT after COVID-19 infection (11).

A sharp increase in cases of COVID-19 infection signifies the importance of an effective vaccine (12). Through different immunogenic pathways, the reliable effect of vaccines is observed *via* randomized trials, with a significant reduction in COVID-19 cases (3). However, side effects of the vaccines have also surfaced. Previously, several cases of SAT caused by COVID-19 vaccines have been reported (13–15). Here, we reported three cases of patients with SAT after receiving the SARS-CoV-2 vaccine to raise awareness on the connection between SAT and the vaccine and discuss the possible pathogenic mechanism.

## Case description

### Basic characteristics

#### Patient 1

A 63-year-old female was admitted to our outpatient department with anterior neck pain and palpitation symptoms. There was no report of thyroid-related disease, autoimmune disease, or preceding upper respiratory system infection in her medical history, and the test for COVID-19 was negative. On 28 April 2021, she received her first dose of the SARS-CoV-2 vaccine (ZF2001 recombinant tandem-repeat dimeric RBD-based protein subunit vaccine) (16), and she developed right-side anterior neck pain, palpitation, and insomnia 2 months after the injection. On 30 June 2021, the patient had aggravating symptoms after the second dose of the vaccine.

#### Patient 2

A 38-year-old female presented to our clinic with left thyroid lobe tenderness. She had a history of chronic hepatitis B and did not report any medical history of thyroid-related and autoimmune disease, and no upper respiratory system infection symptoms were observed before the neck pain. The test for COVID-19 was negative. She received the first dose of the SARS-CoV-2 vaccine (BBIBP-CorV, inactivated virus) on 1 June 2021 (17) and the second on 21 June 2021. Thyroid pain appeared a month after the second vaccine.

#### Patient 3

A 56-year-old female with neck pain and palpitation was admitted to our outpatient department. She did not report any medical history for thyroid-related disease, autoimmune disease, or preceding upper respiratory system infection, and the test for COVID-19 was negative. She had a history of hypertension for 2 years. She received the first dose of the SARS-CoV-2 vaccine (BBIBP-CorV, inactivated virus) on 30 June 2021 (17). Two weeks after the vaccine injection, she had neck pain, fever, and palpitation.

### Clinical data

#### Patient 1

**FIGURE 1:**
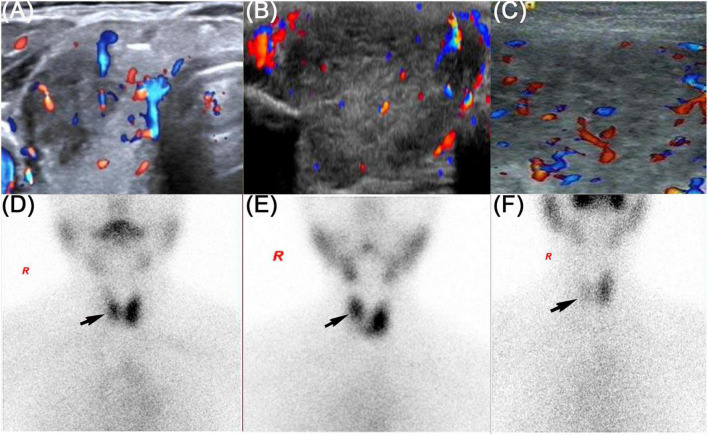
Thyroid ultrasound and thyroid emission computed tomography of three patients. The black arrow showed the radioactive distribution defects in the thyroid lobe. **(A,D)** Refers to patient 1, **(B,E)** refers to patient 2, and **(C,F)** refers to patient 3.

**FIGURE 2:**
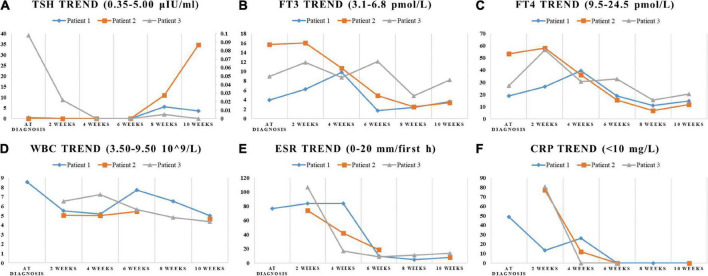
The trend of indicators in laboratory tests. The change of TSH, FT3, FT4, WBC, ESR, and CRP during the SAT period were displayed in **(A–F)**. **(A)** The ordinate axis on the right side is used only for patient 3 due to the large numerical span.

On physical examination, the patient’s heart rate was 95 beats per minute. On palpation, her thyroid gland was sensitive, tender, and enlarged. In her laboratory tests, indicators for thyroid function were within normal range except for elevated thyroglobulin antibody (TgAb). The level of ESR and CRP was high, and white cell blood count (WBC) was normal (Table 1). Thyroid ultrasound (US) demonstrated hypoechoic areas in the enlarged right lobe of the thyroid (Figure 1A). Thyroid emission computed tomography (ECT) showed radioactive distribution defects in the right thyroid lobe (Figure 1D). Therefore, SAT was diagnosed, and celecoxib (200 mg/12 h) and selenium yeast (100 μg/12 h) were started on 13 June 2021. However, the patient’s symptoms were aggravated, and thyroid function began to appear abnormal on 30 June 2021 (Table 2). Thus, the original treatment protocol was replaced with propranolol (10 mg/8 h) and prednisone (10–20 mg twice daily). The symptoms gradually disappeared within a week after the treatment adjustment. Thyroid function tests and inflammatory markers became normal during her follow-up (Figure 2). The patient did not take any drugs during the last follow-up period on 8 September 2021.

**TABLE 1:** Laboratory tests of three cases at diagnosis.

At diagnosis	Patient 1	Patient 2	Patient 3	Reference range
TSH (μIU/ml)	0.543	< 0.005	0.098	0.35–5.00
FT3 (pmol/L)	3.9	15.7	8.9	3.1–6.8
FT4 (pmol/L)	18.9	53.4	27.3	9.5–24.5
rT3 (ng/ml)	0.53	2.59	NA	0.31–0.95
Tg (μg/L)	339.3	182	NA	1.400–78.000
TPO Ab (IU/ml)	9.7	15.2	NA	0.0–34.0
Tg Ab (IU/ml)	19.7	182	NA	0.0–115.0
TR Ab (IU/L)	1.24	6.18	1.75	0.00–1.75
WBC (10^9/L)	8.55	NA	NA	3.50–9.50
ESR (mm/h)	77	NA	NA	0–20
CRP (mg/L)	48.81	NA	NA	< 10

TSH, thyroid stimulating hormone; FT3, free triiodothyronine; FT4, free thyroxine; rT3, reverse triiodothyronine; Tg, thyroglobulin; TPO Ab, thyroperoxidase antibody; Tg Ab, thyroglobulin antibody; TR Ab, thyrotropin receptor antibody, WBC, white cell blood count; ESR, erythrocyte sedimentation rate; CRP, C-reactive protein.

**TABLE 2:** Laboratory tests of three cases at follow-up 1 and last follow-up.

Follow-up 1	Patient 1	Patient 2	Patient 3	Reference range
TSH (μIU/ml)	0.015	0.006	0.022	0.35–5.00
FT3 (pmol/L)	6.2	16	11.9	3.1–6.8
FT4 (pmol/L)	26.4	58.3	56.7	9.5–24.5
rT3 (ng/ml)	0.86	NA	NA	0.31–0.95
Tg (μg/L)	475.5	NA	115.3	1.400–78.000
TPO Ab (IU/ml)	9	NA	15.5	0.0–34.0
Tg Ab (IU/ml)	20.6	NA	554.3	0.0–115.0
TR Ab (IU/L)	1.17	NA	8.28	0.00–1.75
WBC (10^9/L)	5.51	5.05	6.52	3.50–9.50
ESR (mm/h)	84	74	107	0–20
CRP (mg/L)	13.44	77.25	81.01	< 10
Last Follow-up				
TSH (μIU/ml)	3.69	34.69	< 0.005	0.35–5.00
FT3 (pmol/L)	3.6	3.4	8.2	3.1–6.8
FT4 (pmol/L)	14.6	11.7	20.5	9.5–24.5
rT3 (ng/ml)	0.5	NA	1.08	0.31–0.95
Tg (μg/L)	64.37	NA	2.07	1.400–78.000
TPO Ab (IU/ml)	9.6	NA	26.1	0.0–34.0
Tg Ab (IU/ml)	15.1	NA	141.3	0.0–115.0
TR Ab (IU/L)	0.86	NA	4.71	0.00–1.75
WBC (10^9/L)	5	4.69	4.36	3.50–9.50
ESR (mm/h)	8	8	14	0–20
CRP (mg/L)	< 0.5	< 0.5	NA	< 10

TSH, thyroid stimulating hormone; FT3, free triiodothyronine; FT4, free thyroxine; rT3, reverse triiodothyronine; Tg, thyroglobulin; TPO Ab, thyroperoxidase antibody; Tg Ab, thyroglobulin antibody; TR Ab, thyrotropin receptor antibody, WBC, white cell blood count; ESR, erythrocyte sedimentation rate; CRP, C-reactive protein.

#### Patient 2

On physical examination, her heart rate was 79 beats per minute. The thyroid gland was tender on palpation. In her laboratory tests, free thyroxine (FT4) and free triiodothyronine (FT3) were both elevated, and the level of thyroid-stimulating hormone (TSH) was decreased. Thyroperoxidase antibody (TPOAb) and TgAb were within the normal range. Tg and thyrotropin receptor antibody (TRAb) were at a high level. Thyroid ECT indicated radioactive distribution defects in the right thyroid lobe (Figure 1E). Thus, a diagnosis of SAT combined with Graves’ disease was made, and methimazole (5 mg/day) and selenium yeast (100 μg/12 h) were started on 24 July 2021. A month later, the patient felt that her symptoms did not improve and developed a new symptom of palpitation. The US revealed a scattered distribution of hypoechoic areas in the enlarged left lobe of the thyroid (Figure 1B). FT3 and FT4 were higher than the first time, and the level of both ESR and CRP was increased (Table 1). Consequently, the dosage of methimazole (10 mg/day) was increased, and bisoprolol (5 mg/day) was added based on the original treatment protocol. Additionally, we also prescribed celecoxib (200 mg/12 h) instead of prednisone because of the history of hepatitis B. Nevertheless, the patient presented with hypothyroidism on the fifth follow-up assessment, and she was only treated with levothyroxine sodium 50 μg/day (Table 2). On the last follow-up, the patient presented with TSH, FT3, and FT4 in the normal range and mildly increased TRAb value but without any symptoms. She was still treated with levothyroxine sodium 50 μg/day on the last follow-up on December 2, 2021.

#### Patient 3

The patient’s heart rate was 101 beats per minute, and body temperature was 39.5°C on physical examination. FT4 and FT3 were both elevated, and TSH was decreased, which was compatible with thyrotoxicosis. TPOAb was negative, while Tg, TgAb, and TRAb were at high levels. Meanwhile, ESR and CRP were high (Table 1). Thyroid US illustrated the enlarged right lobe of the thyroid (Figure 1C). Thyroid ECT showed radioactive distribution defects in the right thyroid lobe (Figure 1F). Hence, the patient had been considered SAT combined with Graves’ disease according to the symptoms and examination finds. Thereafter, prednisone 10 mg/8 h was started for treatment. The patient was still treated with prednisone 2.5 mg/12 h, and methimazole 2.5 mg/day was added for the diagnosed Graves’ disease (Figure 2). On the last follow-up, the patient presented with TRAb, FT3, and FT4 in the normal range and decreased TSH value but without symptoms. She was still treated with methimazole 7.5 mg/day during her last follow-up period on March 16, 2022.

## Discussion

SAT, also known as De Quervain’s thyroiditis or granulomatous thyroiditis, is a self-limiting inflammatory thyroid disorder characterized by neck pain and general symptoms of thyroid dysfunction. It is most likely caused by viruses, including Coxsackie, influenza, and mumps, and often seen in women (10). All our three cases presented with neck pain or tenderness, acute symptoms of thyrotoxicosis, remarkably elevated inflammatory markers (CRP and ESR), and hypoechoic areas in thyroid US that were further confirmed by thyroid ECT images showing reduced tracer uptake. All the cases were consistent with the diagnosis of SAT (18). Of note, our patients also experienced elevated Tg levels and positive TPOAb or TgAb, which are also consistent with previous reports about SAT (19–21). However, none of our patients had a preceding upper respiratory system infection, and they had no medical history of thyroid-related disease and autoimmune disease. The only common cause of the three patients with SAT is that they got SAT after receiving the SARS-CoV-2 vaccines.

In this report, one patient presented with hypothyroidism. The difference between her and the other two patients is that she did not use prednisone because of her history of hepatitis B. According to existing reports concerning SAT after COVID-19 vaccines, two patients progressed to developing hypothyroidism because of absence of corticosteroid (13, 22). Only one reported that patient’s thyroid function returned to normal without experiencing hypothyroidism after a single use of non-steroidal anti-inflammatory drugs (15). However, there are no clear data on whether glucocorticoids protect from development of hypothyroidism. We suspected that timely treatment of glucocorticoids in patients with SAT might be conducive to improving the disease according to a comprehensive analysis of previous cases. More cases are still needed in the future to confirm our speculation. Interestingly, two of the patients we reported developed elevated TRAb and hyperthyroidism-related symptoms, while thyroid imaging showed the performance of SAT, which indicated that the patients had SAT and Graves’ disease (GD) simultaneously after the vaccines. To the best of our knowledge, this is the first report on SAT in combination with GD after COVID-19 vaccination.

There are currently multiple COVID-19 vaccines, including nucleic acid vaccines (RNA and DNA), adenoviral vectored vaccines, whole-cell inactivated virus vaccines, and subunit protein vaccines (12). However, the mechanism for post-vaccination SAT remains unknown. Some scholars suggest that adjuvants in COVID-19 vaccines lead to occurrence of SAT (14). Adjuvants, especially aluminum hydroxide, are utilized as immunogenicity-enhancing agents to increase the response to vaccination in the general population. Nevertheless, administration of adjuvants may induce autoimmune/inflammatory syndrome as the result of impairing the immunological balance of the host, fostering polyclonal activation of B lymphocytes or other similar etiopathogenetic mechanisms (23). It is noteworthy that several reported cases of SAT after COVID-19 infection were determined (24–28). The mechanism by which SARS-CoV-2 causes SAT has been identified because of the clarified association (15). There is credible evidence to suggest that SARS-CoV-2 uses angiotensin-converting enzyme-2 (ACE-2) combined with the transmembrane protease serine 2 (TMPRSS2), which are highly expressed in the thyroid gland, as the crucial molecular complex to infect host cells (7, 29, 30). In this case, as a target organ, the thyroid gland could be attacked by SARS-CoV-2. Therefore, if heterologous antigens used for vaccination are similar to autologous antigens, the immunologic response induced by heterologous antigens might also react with autologous antigens. Currently, most COVID-19 vaccines are designed to activate the immune response against the SARS-CoV-2 spike protein. The reaction between the SARS-CoV-2 spike protein antibody and tissue proteins, including thyroid tissues, has also been studied implying that viral antigens may trigger inflammation in the thyroid (31). We have a reason to believe that cross-reactivity plays a role in the development of SAT (32, 33). In addition, with the rampant spread of mutant strains of SARS-CoV-2, immunological reactions induced from other sites of virus sequence require further investigation (34). Furthermore, the post-vaccination SAT cases reported now included nucleic acid vaccines, adenoviral vectored vaccines, and whole-cell inactivated virus vaccines (13–15, 22, 35). One of our patients developed SAT after the subunit protein vaccine, which is a valuable supplement to the existing studies. Besides, we calculated the time span of SAT in both our patients and other reported cases. Surprisingly, almost all reported patients with whole-cell inactivated virus and subunit protein vaccination developed SAT about 2 weeks after the second injection. Patients with nucleic acid and adenoviral vectored vaccination developed in a month, which seems consistent with the efficacy of different types of vaccines (12). Only two persons presented with SAT after the first dose of the whole-cell inactivated virus vaccine (14). This phenomenon reminds clinicians that they ought to pay attention to this special period.

In conclusion, we have reported three cases of patients who developed SAT after receiving SARS-CoV-2 vaccines and discussed the possible mechanisms of the disease. The increased incidence of post-vaccination SAT can be observed with the COVID-19 pandemic and accompanying ascending vaccination rate. Early recognition and diagnosis necessitate clinicians’ vigilance. Nevertheless, it should not raise any concern regarding the vaccination, because vaccine-associated SAT is usually of moderate severity and could be easily treated (36).

## Data availability statement

The original contributions presented in this study are included in the article/supplementary material, further inquiries can be directed to the corresponding author.

## Ethics statement

The authors have obtained the patients’ consent and signatures on the informed consent form to publish their cases (including publication of images).

## Author contributions

YH drafted the manuscript. LZ takes full responsibility of the study as a whole, including (if applicable) study design, access to data, and the decision to submit and publish the manuscript. All authors collected the data from the patients and contributed to the writing of the manuscript and helpful discussion.
